# Impact of a 6–12-h delay between ileocolic intussusception diagnostic US and fluoroscopic reduction on patients’ outcomes

**DOI:** 10.1007/s00247-024-05960-2

**Published:** 2024-06-06

**Authors:** Julian Lopez-Rippe, J. Christopher Davis, Rebecca A. Dennis, Summer L. Kaplan, Jorge Delgado

**Affiliations:** 1 Department of Radiology, Children’s Hospital of Philadelphia, 3401 Civic Center Blvd, Philadelphia, PA 19104 USA; 2 Perelman School of Medicine, University of Pennsylvania, 3400 Spruce St, Philadelphia, PA 19104 USA

**Keywords:** Ileocolic intussusception, Fluoroscopic reduction, Failed reduction, Recurrence, Outcomes

## Abstract

**Background:**

Image-guided reduction of intussusception is considered a radiologic urgency requiring 24-h radiologist and technologist availability.

**Objective:**

To assess whether a delay of 6–12 h between US diagnosis and fluoroscopic reduction of ileocolic intussusception affects the success frequency of fluoroscopic reduction.

**Materials and methods:**

Retrospective review of 0–5-year-olds undergoing fluoroscopic reduction for ileocolic intussusception from 2013 to 2023. Exclusions were small bowel intussusception, self-reduced intussusception, first fluoroscopic reduction attempt>12 h after US, prior bowel surgery, inpatient status, and patient transferred for recurrent intussusception. Data collected included demographics, symptoms, air/contrast enema selection, radiation dose, reduction failure, 48-h recurrence, surgery, length of stay, and complications. Comparisons between<6-h and 6–12-h delays after ultrasound diagnosis were made using chi-square, Fisher’s exact test, and Mann–Whitney *U* tests (*P*< 0.05 considered significant).

**Results:**

Of 438 included patients, 387 (88.4%) were reduced in <6 h (median age 1.4 years) and 51 (11.7%) were reduced between 6 and 12 h (median age 2.05 years), with median reduction times of 1:42 and 7:07 h, respectively. There were no significant differences between the groups for reduction success (<6 h 87.3% vs. 6–12 h 94.1%; *P*-value = 0.16), need for surgery (<6 h 11.1% vs. 6–12 h 3.9%; *P*-value=0.112), recurrence of intussusception within 48 h after reduction (<6 h 9.3% vs. 6–12 h 15.7%; *P*-value=0.154), or length of hospitalization (<6 h 21:07 h vs. 6–12 h 20:03 h; *P*-value=0.662).

**Conclusion:**

A delay of 6–12 h between diagnosis and fluoroscopic reduction of ileocolic intussusception is not associated with reduced fluoroscopic reduction success, need for surgical intervention after attempted reduction, recurrence of intussusception following successful reduction, or hospitalization duration after reduction.

**Graphical Abstract:**

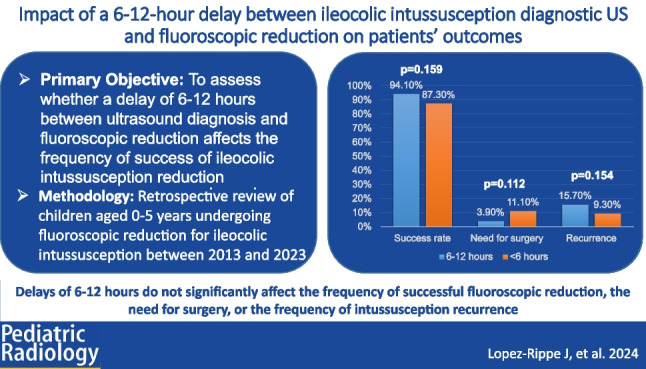

## Introduction

Ileocolic intussusception is a common cause of abdominal pain in children, most commonly affecting children under the age of 5 years [1], that requires prompt identification and treatment to avoid morbidity and mortality [2]. Approximately 90% of ileocolic intussusceptions are idiopathic; however, there are some causes of secondary intussusception due to a lead point such as Meckel’s diverticulum, solid and cystic abdominal mass lesions, blunt abdominal trauma, or intestinal lymphoma [2].

As the classic clinical triad of abdominal pain, palpable abdominal mass, and red currant jelly stool is present in less than 50% of patients [3], ultrasound (US) is the first-line imaging modality for the diagnosis of intussusception, with a sensitivity and specificity near 100% [1–5]. Treatment options include both surgical and image-guided approaches. Image-guided options include fluoroscopic or US-guided air or fluid enema reduction. It is difficult to predict based on US images which cases will be irreducible on air or fluid enema reduction [3, 6–8]. Most ileocolic intussusceptions are initially treated with image-guided reduction [1], which decreases recovery time and the need for additional interventions [1, 9].

Currently, image-guided reduction of ileocolic intussusception is considered a radiologic urgency requiring 24-h radiologist and technologist availability. Failure to perform appropriate treatment may cause bowel ischemia, perforation, peritonitis, and even death [1, 10]. There is contradictory data on whether ileocolic intussusception reduction should be performed emergently (as soon as possible) or urgently (delayed a few hours until optimal support staff is available). Some studies have found no impact on pneumatic or enema reduction success with delays up to 48 h after symptom onset [11–13], while other authors report increased probability of a surgical intervention following delays in image-guided interventions [2, 10, 14, 15].

Our goal is to evaluate whether a delay between diagnosis and fluoroscopic ileocolic intussusception reduction of less than 12 h (to optimize resource and staffing allocation) affects the complication frequency, need for surgical interventions, and patient outcomes.

## Materials and methods

### Participants

This retrospective study was granted an exempt status by our institutional review board and was performed in compliance with the Health Insurance Portability and Accountability Act (HIPAA). We performed a search within our departmental picture archiving and communication system (Softek Illuminate, Overland Park, KS). The search included the terms “fluoroscopy” and “intussusception reduction.” All patients aged 0–5 years who underwent fluoroscopic reduction for ileocolic intussusception at our institution from January 2013 to June 2023 were enrolled for further evaluation. In our hospital, we do not routinely perform US-guided intussusception reductions; thus, these subjects were not part of our inclusion criteria.

Exclusion criteria were as follows: small bowel—small bowel intussusception, self-reduced intussusception, first fluoroscopic intussusception reduction attempt more than 12 h after the diagnostic US, history of prior bowel surgery, inpatient status at the time of intussusception, intussusception diagnosed with a method other than US such as CT or fluoroscopy, and patient transfer to our hospital due to recurrent intussusception. As cases of secondary intussusception (such as abdominal mass or Meckel diverticulum) usually present initially as non-diagnosed cases, we decided to include these cases for further analysis with the aim particularly representative of real-world clinical practice.

Both air and enema fluoroscopic reductions were included for analysis. Only the first episode of ileocolic intussusception was included in the analysis to avoid bias from increased parental awareness of the disease and faster care based on the patient’s known medical history. A 6-h threshold was selected based on an envisioned paradigm wherein fluoroscopic reductions are offered until midnight but then not until 6 am. This should allow optimization of overnight staff coverage, whether in-house or called in.

### Intussusception reduction

At our institution, all cases of suspected ileocolic intussusception follow an established protocol whereby the emergency department clinician orders an emergent diagnostic US. Following a positive US, all patients are evaluated for contraindications to reduction. For all patients undergoing a fluoroscopic intussusception reduction attempt, intravenous access is secured and the patient is monitored. The patient is accompanied to the fluoroscopy suite by nursing staff. The decision to proceed with contrast enema or air intussusception reduction is based on the radiologist’s preference. For air reductions, Shiel’s intussusception air reduction kit with a pressure relief valve set as 120 mmHg is used. For enema reductions, non-diluted iothalamate meglumine (Cysto-Conray®) 17.2% and gravity contrast filling of the bowel is utilized. Following a safety check to confirm patient identity and allergies, procedure explanation to parents, and parental consent, a size-appropriate flex tip enema nozzle is placed in the rectum and appropriately sealed with tape. Fluoroscopic imaging for contrast or air reduction is performed by the radiologist, radiology fellow, and/or radiology resident. The radiation time and patient radiation dose (dose area product, measured in dGy∙cm^2^) are documented for all patients.

### Medical record review

The medical record review was performed by a pediatric radiologist (J.D.) and a physician (J.L.R) with 11 years and 2 years of experience in pediatric radiology research, respectively. Cases were split between the two evaluators with consensus assessment in cases in which additional questions arise. The medical record review included the following data points:

1. Clinical symptoms (based on ED note): (a) length of symptoms in hours before diagnostic US study, (b) bloody stools, and (c) lethargy [14, 16]. Medical charts were also assessed for history of prematurity, cardiac disease, or lung disease with the aim of having a general idea of patient complexity.
2. Diagnostic US study date and time.
3. Fluoroscopic study date and time.
4. Selection of contrast enema or air for fluoroscopic reduction of intussusception.
5. Evaluation of fluoroscopic radiation dose including fluoroscopy time and dose area product (DAP) (dGy∙cm^2^) [17].
6. Fluoroscopic reduction complication (pneumoperitoneum or extraluminal contrast enema extravasation).
7. Failure of reduction defined as persistent intussusception after fluoroscopic reduction attempt. If reduction failure, number of following reduction attempts (defined as subsequent visits to fluoro suite) and success of additional reduction attempts.
8. Recurrence of intussusception defined as visualization of intussusception within 48 h after a successful reduction**.** If intussusception recurred, the number of recurrences was recorded.
9. Surgery required for intussusception treatment. If surgery was performed, presence of bowel perforation, bowel necrosis, or bowel resection was evaluated.
10. Hemodynamic instability or demise.
11. Hospitalization length.

### Statistical analysis

Descriptive analysis for both groups was performed using percentages, means, medians, ranges, and interquartile ranges. We defined two groups: early fluoroscopic reduction (less than 6 h from diagnostic US to fluoroscopic reduction of intussusception) and fluoroscopic reduction (6–12 h from diagnostic US to fluoroscopic reduction of intussusception). To assess differences between these two groups, the chi-square test and Fisher’s exact test were used for categorical variables and the Mann–Whitney *U* test was used for continuous variables. A logistic regression assessing for association between fluoroscopic reduction success and time between diagnosis and fluoroscopic reduction as a continuous variable correcting for age and weight was performed. A post-hoc power analysis for our primary outcome (successful fluoroscopic reduction) was performed. Statistical significance was defined by *P*-values < 0.05. All analyses were performed using the open and free statistical program Jamovi 2.3.

## Results

**Fig. 1:**
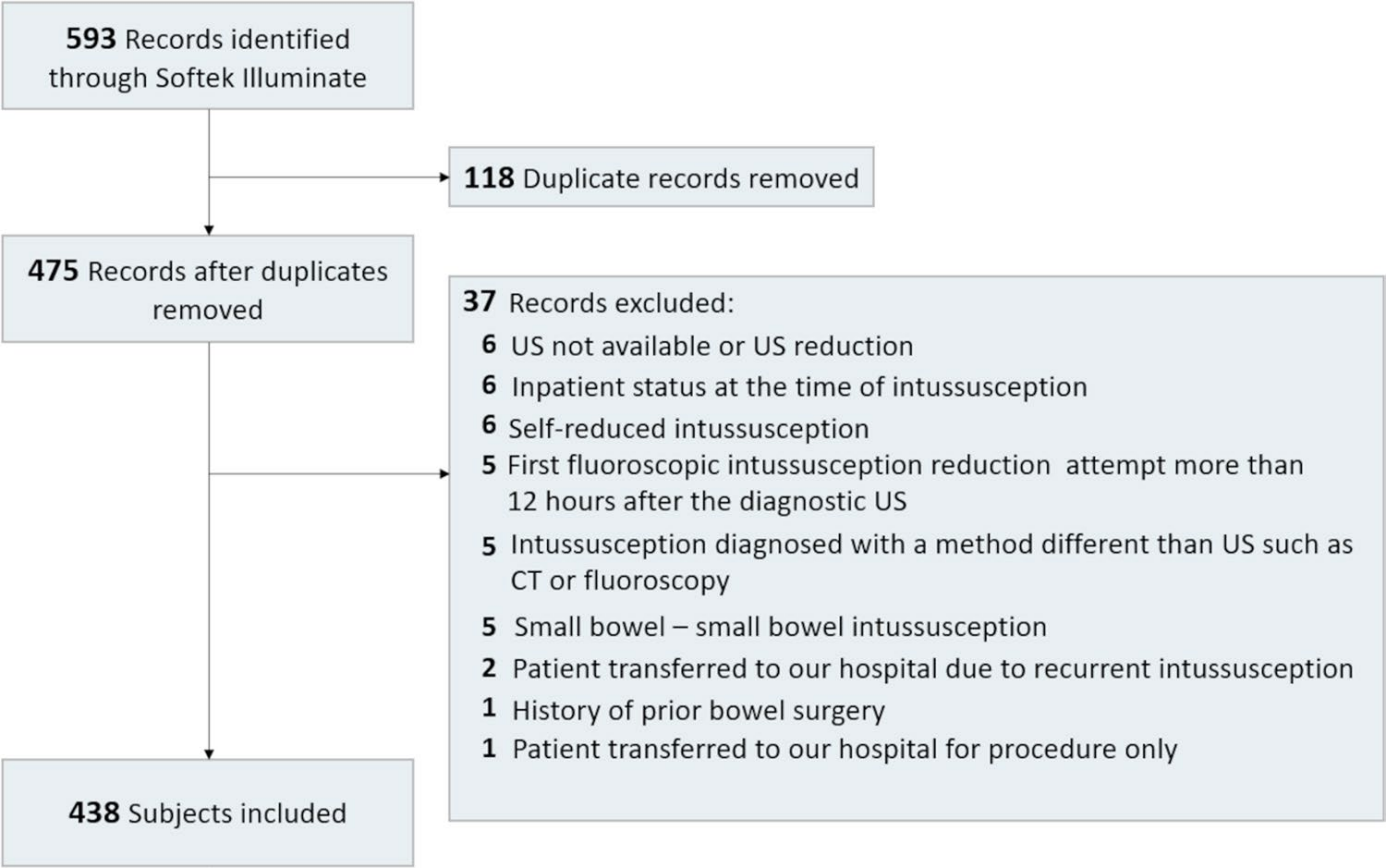
Flow diagram of patients’ selection

A total of 438 patients met the inclusion and exclusion criteria (Fig. 1; Table 1). Of these, 387 patients (88.4%) underwent fluoroscopic reduction of ileocolic intussusception less than 6 h after diagnostic US, and 51 patients (11.6%) received fluoroscopic reduction of intussusception between 6 and 12 h after US. The median age was significantly higher in the 6–12-h group compared to the < 6-h group (median 2.05 years vs. 1.4 years, *P*-value = 0.007) (Table 1). A significantly higher proportion of patients in the 6–12-h group were referred from an outside hospital compared to the < 6-h group (98% vs. 34.6%, *P*-value < 0.001). There were no significant differences between the groups in terms of sex, length of symptoms, frequency of bloody stools, history of prematurity, cardiac disease, lung disease, or use of pre-procedural antibiotics.

**Table 1:** Characteristics of patients undergoing fluoroscopic intussusception reduction between 6 and 12 h and less than 6 h after diagnostic US

Time between US and fluoroscopic reduction	Between 6 and 12 h (*n* = 51)		Less than 6 h (*n* = 387)		*P*-value
Median time (IQR)	7:07 h (1:28 h)		1:42 h (1:56 h)		
Gender (male/female)	36/15 (70.6%/29.4%)		233/154 (60.2%/39.8%)		0.152*
Median age in years (IQR)	2.05 (1.34)		1.4 (1.15)		0.007***
Median weight in kg (IQR)	12.1 (4.5)		10.7 (5.15)		0.007***
Referred from outside hospital	50 (98%)		134 (34.6%)		< 0.001*
Length of symptoms	< 24 h	19 (37.3%)	< 24 h	150 (38.8%)	0.244*
	24–48 h	9 (17.6%)	24–48 h	103(26.6%)	
	> 48 h	23 (45.1%)	> 48 h	134 (34.6%)	
Bloody stools	12 (23.5%)		69 (17.8%)		0.92*
Lethargy	0		5 (1.3%)		1.00**
Secondary intussusception	2 (3.9%)		12 (3.1%)		0.672**
History of prematurity	1 (1.96%)		12 (3.1%)		1.00**
Cardiac disease	1 (1.96%)		5 (1.29%)		0.526**
Lung disease	0		16 (4.13%)		0.236**
Pre-procedural antibiotics	0		4 (1%)		1.0**

^*^Chi-square test

^**^Fisher’s exact test

^***^Mann–Whitney *U* test

The frequency of success of fluoroscopic reduction after a single attempt was not significantly different between the 6–12-h and < 6-h groups (94.1% vs. 87.3%, *P*-value = 0.16) (Table 2). Based on the observed frequency of reduction success, post-hoc power analysis demonstrated that we had 24% power to identify a significant difference in reduction success between groups. Logistic regression showed no association between ileocolic intussusception reduction success and time from ultrasound diagnosis to fluoroscopic reduction correcting for age and weight (*P*-value = 0.32). Table 3 displays the distribution of fluoroscopic reduction success frequency in hourly increments. A single complication consisting of pneumoperitoneum during air fluoroscopic reduction was observed. The subject was an 8-month-old with 5 days of symptoms, bloody stools, and a 6.3-h delay in between ultrasound diagnosis and fluoroscopic reduction.

**Table 2:** Differences between patients undergoing early (less than 6 h) versus mildly delayed (6–12 h) fluoroscopic reduction of intussusception

	Between 6 and 12 h (*n* = 51)		Less than 6 h (*n* = 387)		*P*-value
Successful fluoroscopic reduction (after single attempt)	48 (94.1%)		338 (87.3%)		*P* = 0.159*
Subjects requiring multiple attempts to achieve reduction	2 (3.9%)		11 (2.8%)		*P* = 0.655**
Pneumoperitoneum or extraluminal contrast during fluoroscopic reduction	1 (2%)		0		*P* = 0.116**
Enema or air reduction	Air	30 (58.8%)	Air	227 (58.7%)	1.00**
	Contrast	21 (41.2%)	Contrast	153 (39.5%)	
	Both	0 (0%)	Both	7 (1.8%)	
Recurrence of intussusception	8 (15.7%)		36 (9.3%)		*P* = 0.154*
Surgery required to reduce intussusception	2 (3.9%)		43 (11.1%)		*P* = 0.112**
Surgery for comorbidity (i.e., resection of leading mass/Meckel diverticulum)	1 (2%)		6 (1.6%)		*P* = 0.290**
Bowel necrosis	1 (2%)		8 (2.1%)		*P* = 0.357**
Median fluoroscopy time (min) (IQR)	1.90 min (2.5)		1.8 min (2.4)		*P* = 0.828***
Median hospitalization length after fluoroscopy (IQR)	20:03 h (20:36 h)		21:07 h (20:49 h)		*P* = 0.662***

^*^Chi-square test

^**^Fisher’s exact test

^***^Mann–Whitney *U* test

**Table 3:** Distribution of fluoroscopic reduction success frequency in hourly increments

Time from diagnostic US to fluoroscopic reduction	Fluoroscopic intussusception reduction time group	Successful fluoroscopic reduction	Unsuccessful fluoroscopic reduction
0–1 h	< 6 h	43 (87.8%)	6 (12.2%)
1–2 h		154 (88.5%)	20 (11.5%)
2–3 h		50 (78.1%)	14 (21.9%)
3–4 h		29 (96.7%)	1 (3.3%)
4–5 h		29 (82.9%)	6 (17.1%)
5–6 h		33 (94.3%)	2 (5.7%)
6–7 h	6–12 h	21 (91.3%)	2 (8.7%)
7–8 h		14 (93.3%)	1 (6.7%)
> 8 h		13 (100.0%)	0 (0.0%)
**Total**		386 (88.1%)	52 (11.9%)

The proportion of patients requiring multiple attempts to achieve reduction and the overall frequency of successful reduction after multiple attempts were also comparable between the groups (Table 2). There were no significant differences in the rates of recurrence, need for surgery, presence of bowel necrosis, surgery for comorbidities, median fluoroscopy time, or median hospitalization length after the procedure (Table 2).

## Discussion

In this retrospective study, we found no significant differences between patients undergoing early versus delayed fluoroscopic reduction of ileocolic intussusception in terms of frequency of successful reduction, need for a surgical intervention after attempted reduction, recurrence of ileocolic intussusception following successful reduction, or hospitalization length after reduction.

Reported success of fluoroscopic reduction of ileocolic intussusception ranges between 74 and 98% [1, 2, 9, 12, 14, 16]. Our fluoroscopic reduction success frequency was in this range for both groups. Ileocolic intussusception can be a serious condition, with mortality approximating 0.2% and about 3.5% of patients requiring an ICU admission in children’s hospitals in the USA [1]. The mortality risk tends to be associated with comorbidities and surgical interventions. Shapkina et al. found a low perforation of 0.4% with fluoroscopic reduction [9]. In our study, a low frequency of adverse events, such as bowel necrosis or resection, was observed. These events occurred in 2% of cases undergoing fluoroscopic reduction between 6 and 12 h and in 2.1% of cases undergoing fluoroscopic reduction less than 6 h from diagnosis.

Our results are concordant with similar research recently published by Williams et al. [13]. Those authors assessed the morbidity and frequency of ileocolic intussusception reduction success rate in patients with different durations of delay between intussusception diagnosis and fluoroscopic enema reduction. In their dataset, 41 patients had a fluoroscopic reduction delay between 3 and 6 h and 11 patients a fluoroscopic reduction delay between 6 and 8 h. There was no significant difference in reduction efficacy or complication frequency.

In a separate study, Lampl et al. compared the time from ileocolic intussusception diagnosis to image-guided reduction between patients that required and did not require surgery. They found that the median time between diagnosis and image-guided reduction was higher in patients who required a surgery (median time 17.9 h) versus patients who did not (median time 7.0 h) [10]. While we observed no significant difference in the need for surgical reduction between our groups, the delayed intervention group in our study had a more similar interval to image-guided intervention as the group that did not go to surgery in the study by Lampl et al. (7:07 h vs. 7 h). This raises the possibility that more prolonged delays (> 12 h) between diagnosis of ileocolic intussusception and initial image-guided intervention may be associated with less good outcomes.

Liu et al. compared patients with a history of symptoms for more than 48 h to patients with symptoms for less than 48 h. They found no differences in success, recurrence, or perforation frequencies. They also found that the presence of bloody stools had a non-significant trend toward less successful reductions [12]. This conflicts with previously published articles. Fike et al. found that failed reduction was more likely if patients had symptoms for more than 24 h before presentation or if patients had bloody stools or lethargy at the time of presentation. They also found that the chances of a successful reduction were also reduced by a more distal colonic extent of the intussusception into the transverse or descending colon [14]. According to Lehnert et al., surgical intervention was more likely in patients presenting after 24 h of symptoms [2]. Zouari et al. found that duration of symptoms longer than 48 h and fever at admission were risk factors for intussusception recurrence [18]. In reviewing approximately 470 charts for this study, we found that it is difficult to assess the precise onset of patients’ symptoms due to the insidious initiation of symptoms. Most patients presenting with ileocolic intussusception tend to be non-verbal or have limited verbal abilities at the typical age of presentation. There is also an association between ileocolic intussusception and other infections (e.g., upper respiratory tract infections) which may act as a confounding variable. For these reasons, we believe it would be difficult to implement a reliable triage of the patients based on duration of symptoms. Further studies are needed to explore the association between early fluoroscopic reduction and outcomes in patients with bloody stools or lethargy at the time of presentation.

While our study demonstrates that a short delay in fluoroscopic reduction of ileocolic intussusception may not significantly negatively impact clinical outcomes, it is important to acknowledge the potential costs and challenges associated with prolonging observation or hospital admission. Delaying the procedure could potentially lead to increased duration of patient discomfort, parental anxiety, and increased healthcare costs related to extended monitoring and hospital stay. These all need to be explored as balancing measures in analyses seeking to optimize staffing for ileocolic intussusception reduction. A short delay to initial fluoroscopic (or US) reduction attempt could have multiple advantages for the planning of care. Even in institutions with 24/7 pediatric radiologist coverage such as ours, we have a single radiologist reading all cases during the night. Having this radiologist perform a procedure delays other patients’ care on the ED and inpatient services. Fluoroscopy technologist coverage is also an issue to consider. In our institution, we are required to call the on-call technologist. In times of short staffing, a lighter call schedule could be beneficial for recruitment and talent retention. It is also worth considering that the surgery and anesthesia teams also have lower resources at certain times of the day, making the response to any possible complication during a fluoroscopic reduction more difficult. Further research is needed to assess the cost-effectiveness and patient satisfaction associated with different timing strategies for ileocolic intussusception reduction, as well as to evaluate the impact on healthcare staff and resource allocation.

Our study found no significant difference in median length of hospitalization after reduction between patients undergoing early versus delayed fluoroscopic reduction of ileocolic intussusception. After reduction, the median stay was around 20 h in both groups. This aligns with previous studies showing that successful reduction allows discharge within 24 h [19]. While some studies suggest that very early reduction within 6 h optimizes outcomes [11], our data provides reassurance that delaying up to 12 h does not negatively impact hospitalization time.

Although there are US findings associated with a lower reduction success frequency [15], such as enlarged mesenteric nodes, ascites, left-sided intussusception, and trapped fluid, it is difficult to predict which cases will have a successful air or contrast reduction based on diagnostic US imaging. Kong et al. found that absent flow on Doppler imaging was associated with a reduced frequency of successful reductions; nevertheless, the reduction attempt was successful in 31% of cases with absent flow [6]. Koumanidou et al. compared the reduction success frequency between patients with enlarged lymph nodes within the intussuscipiens (at least two lymph nodes with one measuring 11 mm or more in the long axis) with patients that did not. They found that reduction was successful in only 46% of patients with enlarged lymph nodes within the intussuscipiens compared to 81% in patients that did not [7]. Even in patients with a known lead point, fluoroscopic intussusception reduction can be used with a success frequency of 60% to temporize the need for emergent surgery [8]. Given the non-negligible frequencies of success in patients with these US findings, for clinical practice, none of these US findings is a true contraindication to attempt a fluoroscopic reduction. In prior studies, spontaneous reduction of intussusception happened in 2.4 to 11% of cases [2, 20].

There are several limitations of this study. Due to the high frequency of reduction success and the rarity of complications with fluoroscopic reduction of ileocolic intussusception, this study is underpowered to detect small differences between the groups. Most cases in the delayed reduction group (6–12-h group) were transferred from outside institutions nearby our main campus which may have unidentified influences on outcomes of interest. It is also important to consider that children who appeared in poorer condition may have had priority or more effort placed by the referring institutions to expedite transportation to our main campus. Thus, prospective research with close follow-up is required to further assess the outcomes of this practice modification.

## Conclusion

A delay of 6 to 12 h between diagnostic US and ileocolic intussusception reduction is not associated with a reduced fluoroscopic reduction success, a need for surgical intervention after attempted reduction, recurrence of intussusception following successful reduction, or increased hospitalization length after reduction. Cautious attempts to complete intussusception reduction when more optimal resources are available could be considered in the future.

## Data Availability

The authors declare that they had full access to all of the data in this study and the authors take complete responsibility for the integrity of the data and the accuracy of the data analysis.
